# Physical Exploration and Diagnosis of Diseases Affecting the Respiratory Organs

**Published:** 1856-10

**Authors:** 


### BOOK NOTICES.

*Physical Exploration and Diagnosis of Diseases Affecting the Respiratory Organs.* By AUSTIN FLINT, M.D., Professor of the Theory and Practice of Medicine, in the University of Louisville, &c. Philadelphia, Blanchard & Lea, 1856.

This volume has been on our table for several months, waiting a notice—waiting not from want of merit in itself, but for want of time on our part to give it such an examination and review as it deserves. This time we have not yet found, and we bespeak the indulgence of both author and publishers.

Dr. Flint is one of the most industrious and energetic men in the medical profession of this country. His previous contributions to our medical literature, have won for him both American and European reputation, and we assure our readers that the present volume is full of valuable and interesting matter. The work is divided into two parts: the first of which treats of Physical Exploration of the Chest; the second, of Diseases Affecting the Respiratory Organs. We present the following summary of the sounds heard on percussion, both for the purpose of showing the doctrines of the author, and for their intrinsic value:—

SUMMARY.

"The abnormal sounds developed by percussion are distinguished from each other, and from the normal thoracic resonance, by variations in timbre, or quality, in intensity, in pitch, and in duration. For practical purposes it suffices to arrange them into divisions based on differences in intensity and in quality; variations in pitch and duration furnishing incidental characters. Thus arranged, the several classes of abnormal sounds are as follows: 1, exaggerated vesicular resonance; 2, diminished resonance; 3, absence of resonance; 4, tympanitic resonance."

1. Exaggerated vesicular resonance is characteristic of vesicular emphysema. It is highly distinctive of that affection, unless the distension of the cells and expansion of the thoracic walls be very great, when the sonorousness may be diminished. Exaggerated resonance from emphysema retains the vesicular quality distinctive of normal resonance, but this quality may be diminished more or less, and the sound approximate in timbre to the tympanitic. In proportion as the latter alteration takes place, the pitch is raised."

"2. Diminished resonance, or dulness, occurs, as a general rule, when a thin stratum of liquid removes the lung a short distance from the chest; when the pulmonary substance is condensed by pressure of liquid effusion within the pleural sac, or, more rarely, by fluids in the bronchial tubes, by serous effusion within the cells or areolar tissue, and by vascular engorgement; by tumors encroaching on the thoracic space, and by deposit of solid products within the lungs, viz,, coagulable lymph, tubercle, carcinomatous matter and a bloody clot; *cœteris paribus*, the degree of dulness is in proportion to the extent to which the air-cells are compromised, and the relative quantity of air to the solid parts reduced. Important exceptions to this rule are observed. In a large proportion of cases of pleural effusion, the percussion-sound above the level of the liquid, for a variable period during the progress of the disease, is exaggerated, and in its character tymanitic. The same is true of the percussion-sound over the healthy lobe on the side in which the lower lobe is solidified in pneumonitis. A tympanitic resonance is propagated from the stomach and intestines in cases of soidlification of the lower lobes, more especially the left lobe. It accompanies also, sometimes, partial solidification from tubercle, or other deposits at the summit of the chest. Whenever the sound becomes dull, the pitch is raised, and the duration shortened. The pitch is also higher when it becomes tympanitic. The diseases in which diminished resonance occurs, with the exceptions just stated, are pleurisy and hydrothorax, above the level of the liquid; pneumonitis; œdema of the lungs; great congestion; pulmonary apoplexy; carcinoma, and tuberculosis."

"3. Absence of resonance, or flatness, is occasioned by an accumulation of liquid in the pleural sac, and exists below the level of the liquid; sometimes by complete solidification in pneumonitis, and by tumors, or morbid growths. An increased sense of resistance, under these circumstances, is marked."

"4. Tympanitic resonance embraces all abnormal sounds (exclusive, of course, of flatness, which is, strictly speaking, absence of sound), which are non-vesicular. It exists in the most marked degree in cases of pneumo-hydrothorax. But in this affection, if the walls of the chest are distended so as to be made quite tense, the sound may become dull, although in character still tympanitic. The sound transmitted from the stomach or intestines, when percussion is made over solidified lung, is purely tympanitic. The sonorousness sometimes existing overcon densed lung, or lung solidified by morbid deposits, at the summit of the chest, is also more or less tympanatic. A tympanitic resononce may also be developed by percussion over tuberculous excavations. In the latter case it is circumscribed in extent. Tympanitic resonance, under these circumstances, occasionally presents a ringing metalic intonation, and it is then called *amphoric resonance.* This modification is sometimes observed when sonorousness exists over solidified lung. Another modification is a *cracked-metal sound* (*bruit de pot fele*), sometimes produced by percussing over a cavity of considerable size, superficially situated, having rigid walls, and communicating freely by several orifices with the bronchial tubes. The same peculiar sound, however, has been, repeatedly observed in children at the summit of the chest, being caused by the forcible expulsion of the air from the bronchial tubes."

The chapter on Auscultation contains full directions for the examination of the chest, and a careful and accurate description of the different sounds that may be heard: first, in a healthy chest; and, secondly, in one affected by disease. The different sounds are carefully analyzed and traced to their physical causes. We observe a change in the nomenclature, so far as the term *rude* respiration is concerned—the author substitutes the term *bronch-vesicular* as indicating both the character and source of the sonnds. It is described as follows:—

"*Inspiration* presenting vesicular and tabular qualities mixed; shortened in duration; pitch raised; intensity variable; sometimes alone present. *Expiration* oftener present; frequently existing alone; prolonged; occurring after an interval; pitch higher than that of inspiration, and oftener more intense."

The chapter on the condition of the Physical Signs is so important, that we quote the greater part of it, premising that a careful study of the previous chapters is requisite in order to fully understand it:—

SIGNS CORRELATIVE TO THOSE FURNISHED BY PERCUSSION,

"1. *Exaggerated Viscular Resonance.*—Occuring in consequence of the activity of the lung on one side being supplementarily increased, the correlative sign pertaining to auscultation is an exaggerated vesicular murmur. Under such circumstances, however, these signs are not intrinsically morbid. They are physiological phenomena exaggerated, but not to a point to be in themselves pathological, and they denote intra-thorcie disease, not at the portion of the chest corresponding to the situation where they are observed, but, inferentially, at another part, and generally on the opposite side. A correlative sign obtained by inspection and mensuration is increased extent of the respiratory movements. The pathological relation of exaggerated resonance to emphysema is more direct and important. The morbid condition in this affection consists in an abnormal accumulation of air, generally within the pulmonary cells, in some rare instances in the interlobular and sub-serous areolar tissue. The correlative sign derived from auscultation is directly the reverse of that in the previous instance, viz., diminution of the respiratory murmur, amounting sometimes to suppression. This combination is highly significant. Other auscultatory signs are frequently associated, but they are incident, not purely to the emphysema, but to coexisting affections, especially bronchitis. This remark applies to the bronchial rales so often present in cases of emphysema. Associated signs, determined by inspection are thoracic enlargement, general or local, corresponding to the extent of the emphysematous dilatation; diminished respiratory movements; obliteration of intercostal depressions; diminished obliquity of the upper ribs, divergence of the lower, and convergence of the upper ribs; if the emphysema be general. The relation of exaggerated resonance to emphysema is the rule; but occasional exceptions are present in cases of great tenseon of the thoracic walls from the pressure of an over distended lung. In these exceptional instances the resonance may be diminished in place of being exaggerated. The viscular quality of resonance in cases of emphysema is rarely if ever lost, but it is more or less diminished. It is vesiculo-tympanitic. In proportion as the intensity of resonance is diminished by tension, the vesicular quality is impaired, and the tympanitic predominates.

"Exaggerated percussion-resonance incident to the temporary emphysematous condition which sometimes obtains in bronchitis, pulmonary catarrh, and bronchial spasm, involves, as correlative signs, the adventitious sounds which pertain to these affections, viz., the dry and moist bronchial rales.

"2. *Diminished Vesicular Resonance.*—In the exceptional instances of emphysema in which this modification of percussion-resonance occurs, the correlative signs will, of course, be the same which, in the majority of instances of that affection, are combined with exaggerated resonance.

"Commonly the affections to which diminution of resonance is incident are those involving either liquid pleural effusion, viz., pleurisy and hydrothorax; or increased density of lung from deposit of liquid or solid matter, viz., pneumonitis, tuberculosis, œdema, pulmonary apoplexy, carcinoma, etc. The correlative signs in these two classes of affections are far from identical; nor are they uniform in the different affections included in the same class.

"In pleuritic effusion sufficient to diminish but not abolish the vesicular resonance, correlative auscultatory signs are, diminished respiratory murmur, and in some instances ægophony. Correlative signs determined by palpitation are, diminished or suppressed vocal vibration, and increased force of resistance to pressure.

"In solidification from pneumonitis and tuberculosis, the correlative auscultatory phenomena, in the majority of instances, are more or less of the characters of the broncho-vesicular, or of the bronchial respiration, together with exaggerated vocal resonance, or bronchophony, and increased vocal fremitus. Exceptional instances are not very infrequent in which, instead of these signs being associated, the respiratory sound is abolished and the vocal resonance and fremitus not increased. The latter constitute the rule, rather than the exception, in the other affections involving abnormal density of lung, viz., œdema, pulmonary apoplexy, carcinoma, etc.

"A correlative sign in cases of œdema, and, less constantly, in cases of pulmonary apoplexy, is the sub-crepitant rale. The crepitant rale is generally associated with diminished percussion-resonance in pneumonitis, but the converse does not hold good to the same extent; in other words, the crepitant rale often appears before the percussion-resonance is sensibly diminished.

"Diminished respiratory movements may be combined in all the affections named, but oftener in pneumontis and tuberculosis. Increased force of resistance on pressure, and diminished elasticity, is a correlative sign common to all the varieties of solidification.

"3. *Absence of Resonance.*—The anatomical conditions giving rise to diminished resonance may be sufficient to abolish it, rendering the percussion-sound flat, Absolute flatness being in the great majority of instances due to the presence of a considerable quantity of liquid in the pleural cavity, the correlative auscultatory signs are absence of respiratory sound, and of vocal resonance, with notably diminished elasticity of the thoracic walls. This combination of signs is highly diagnostic; yet the rule is not without exceptions, diffused bronchial respiration being associated with flatness in some cases of large effusion. Absence of vocal fremitus is another correlative sign. If the amount of effused liquid be great, inspection and mensuration furnish important associated signs, viz., enlargement of the chest; obliteration of the hollows between the ribs; divergence of the lower and convergence of the upper ribs; comparative immobility; elevation of the shoulder; widening of distance between the nipple and the median line; depression of the liver, and removal of the heart from its normal position. Fluctuation is occasionally appreciable. This collection of signs incident to enlargement of the chest, may, however, to a considerable extent, be reversed, in combination with flatness on percussion over the greater part of the chest. Absorption of the liquid effusion, inducing contraction, may take place, but not sufficiently to permit a return of percussion-resonance, with reappearance of respiratory sound, vocal resonance, and fremitus. Then, in connection with diminished size of the affected side, there will be convergence of the lower ribs, and divergence of the upper; depression of the shoulder, and narrowing of the distance between the nipple and the median line. Obliteration of the intercostal hollows and comparative immobility will be likely to continue.

"Flatness on percussion may accompany abundant tuberculous deposit, the second stage of pneumonitis, and other affections involving abnormal density of the pulmonary substance. The facts pertaining to correlative signs, which have been stated under the head of diminished resonance, or dulness, incident to pulmonary solidification, will be equally applicable, and need not be repeated.

"4. *Tympanitic Resonance.*—The signs associated with the different varieties of tympanitic resonance differ widely, according to the diversity of anatomical conditions represented. In the affection which presents more than any other a resonance purely tympanitic, strongly marked and diffused, viz., pnemohydrothorax, the correlative phenomena derived from auscultation are, the characteristic vocal, tussive, and respiratory sign, metallic tinkling; feebleness or extinction of the vesicular murmur; blowing and amphoric respiration, occasional and irregular; absence of vocal resonance. Inspection and mensuration furnish the group of appearances incident to enlargement from liquid effusion. Palpitation discloses absence or marked diminution of the normal vocal fremitus. Succussion develops the sign incident almost exclusively to this affection, viz., splashing.

"Tympanitic resonance, circumscribed in extent at the summit of the chest, sometimes metallic or amphoric, and occasionally presenting a cracked-metal modification — these circumstances denoting its connection with a spacious pulmonary cavity, superficially situated, with rigid walls and free from liquid contents—exists in combination with cavernous respiration, presenting sometimes an amphoric intonation, alternating with gurgling; occasionally splashing, with the act of coughing, and metallic tinkling; pectoriloquy in some instances; local depression or flattening at the summit of the chest, and deficient expansibility.

"Occurring, as an exception to the general rule, over lung solidified by inflammatory exudation, it is combined, of course, with the various phenomena incident to that anatomical condition.

"When presented in pleurisy, situated above the level of the liquid effusion, and also over the healthy lung in cases of pneumonitis, it cannot be said to have any definite correlative signs, irrespective of those which pertain to the diseases of which it is an incidental feature.

"SIGNS CORRELATIVE TO SOUNDS FURNISHED BY AUSCULTATION.

"1. *Increased Intensity of Vesicular Murmur.*—Proceeding always from hyper-activity of respiration induced supplementarily in a portion of the pulmonary apparatus, the correlative signs are exaggerated percussion-resonance, and increased respiratory movements. The remarks made under the head of Exaggerated Vesicular Resonance are here equally appiicable.

"2. *Diminished Intensity of Vesicular Murmur.* — The phenomena associated with this sign are quite opposite in their character, corresponding to differences in morbid conditions which present a contrast equally striking.

"Abnormal feebleness of the vesicular murmur may be due to the removal of the lung at a certain distance from the thoracic wall. This removal is caused by the presence, in some cases, of air or gas; in others, by a stratum of liquid or solid matter, and sometimes by air and liquid together, in the pleural cavity. In the first instance, a correlative percussion-sign is tympanitic resonance; in the second iustance, it is absence of resonance, or flatness; and in the third instance, both are conjoined, *i. e.*, tympanitic resonance exists above the level of the liquid, and flatness below this level. The presence of air and liquid, constituting pneumo-hyyrothorax, is, however, very rarely characterized by simple feebleness of the respiratory sound; either the latter is abolished, or presents the cavernous or amphoric modification. Correlative signs incident to this affection are metallic tinkling and a succussion-sound. Diminution of the respiratory motions, of vocal resonance, and fremitus, are common to the morbid conditions just mentioned.

"Again, feebleness of respiration, without change in quality or rhythm, occurs in a certain proportion of cases of solidification from tubercle, inflammatory exudation, œdema, etc. On the other hand, it is incident to emphysema, bronchitis, and partial obstruction at any point in the air-passages. In these two classes of morbid conditions the correlative percussion-signs are precisely reversed. In the first class it is combined with diminished resonance, or dulness; in the second, the clearness of the percussion-sound is either undiminished or exaggerated. The anatomical condition in both instances is marked by the combination. Exclusive of the cases in which the lung is removed by liquid or solid matter, air, or gas, from the thoracic wall, feebleness of the respiratory murmur, combined with dulness on percussion, as the rule, denotes increased density of the pulmonary organ; combined with normal resonance, it indicates that the density is neither increased nor diminished; combined with exaggerated resonance, it is evidence of the abnormal rarefaction of the lung, pertaining to emphysema and some cases of bronchitis. Other signs existing in combination serve to establish the distinction as respects the anatomical condition. In cases of solidification, in which the effect on the respiratory sound is simply to diminish its intensity, the vocal resonance may be exaggerated, and even bronchophony may be present. In cases of rarefaction, this occurs only as rare exceptions to the general rule. The same remark will apply to vocal fremitus. Diminished respiratory motions may accompany both anatomical conditions. Enlargement of the chest, and its attendant phenomena, determined by inspection, mensuration, and palpitation, pertain to emphysema. Diminished elasticity of the thoracic walls belongs to the former anatomical condition (increased density); increased elasticity to the latter (rarefaction).

"3. *Suppressed Respiration.*—Abolition of the sound of respiration, occurring in connection with the same diversity of morbid conditions as diminished intensity of the respiratory murmur, presents similar combinations with other signs.

"Accumulation of liquid or gas, or both air and liquid, within the pleural sac, in sufficient quantity to render respiration inaudible, gives rise, in the first instancn, to flatness on percussion; in the second instance, to tympanitic resonance; and in the third instance, to tympanitic resonance above and flatness below the level of the liquid. Diminished respiratory movements, together with absence of vocal resonance and fremitus, are common to the three morbid conditions, and, in addition, first in the order of time, are presented the phenomena attending enlargement of the chest, which need not be again enumerated; and, second, the reversed phenomena following absorption of the fluid, sufficient to induce contraction, but not to permit re-appearance of the respiratory sound.

"In the cases of solidification from tubercle, inflammation, œdema, etc., in which suppression occurs, it is combined with notable dulness on percussion, as the rule, and with a clear tympanitic resonance, as an exception to the rule. Exaggerated vocal resonance, or bronchophony, and increased vocal fremitus, may exist in combination, together with diminished respiratory movements. On the contrary, in the cases of emphysema, in which the respiratory sound is lost, exaggerated percussion-resonance, with more or less of the tympanitic quality (vesiculo-tympanitic resonance), is the associated sign as the rule, dulness being observed as an exception to the rule, in some instances in which the tension of the thoracic wall, from distension, is very great. In the former anatomical condition (solidification), the elasticity of the parietes of the chest is notably diminished; in the latter (rarefaction), the elasticity is increased. In connection with the suppressed respiratory sound incident to emphysema, the vocal resonance and fremitus are not exaggerated, save in some rare exceptional instances, the reverse being true, as already mentioned, of solidification.

"4. *Bronchial Respiration.*—The bronchial respiration represents solidification of lung, except when it occurs in connection with dilated bronchial tubes, increased density of the pulmonary parenchyma, in the latter case being superadded. The correlative signs, therefore, are those which have direct relation to pulmonary solidification, as it exists more especially in tuberculosis and pneumonitis, the bronchial respiration being much oftener present and more strongly marked in these than in other affections in which the density of the lung is increased, viz., œdema, extravasation of blood, etc. The group of signs has been already given in connection with diminished vesicular percussion-resonance, and diminished or suppressed vesicular murmur, when these signs are due to the same anatomical condition, *i. e.*, solidification. The associated signs, when the bronchial respiration exists, are much more uniform than those presented in combination with dulness or flatness on percussion, or with suppressed or diminished respiration, owing to the fact that the anatomical condition represented, in the vast majority of instances, by the bronchial respiration is the same, while the signs last mentioned are incident to anatomical conditions different, and indeed opposite, in their character. Dulness on percussion, exaggerated vocal resonance, or bronchophony, increased vocal fremitus, diminished respiratory movements, increased force of resistance to pressure, are the signs sustaining a correlative relation to the bronchial respiration.*

"5. *Broncho-Vesicular Respiration.* — Representing slight or moderate increase of the density of lung; the correlative relations of this modification of the respiratory sound are essentially similar to those belonging to the bronchial respiration. The difference is, the signs which may be associated are less frequently present, and, when present, are less marked. Dulness on percussion is comparatively slight, and may not be appreciable; the vocal resonance and fremitus may not be obvious, and, if apparent, are weak; the respiratory movements are, perhaps, not sensibly diminished, or, if so, in a small degree; and impairment of the elasticity of the thoracic walls is either not determinable, or feeble.

"6. *Cavernous and Amphoric Respiration.* — Correlative cavernous signs form a group, each preserving always its significance, and not occurring in connection with other anatomical conditions. Actually, however, they are rarely combined, and, indeed, it is impossible for all of them to be present simultaneously, since some can only be produced when the cavity is empty, and others only when it is more or less filled with liquid. The correlative signs requiring an empty space, are the cavernous respiration, pectoriloquy, and circumscribed tympanitic percussion-resonance, inclusive of the metallic modification, and the cracked-metal sound. The correlative signs requiring the presence of liquid, are, circumscribed dulness on percussion, gurgling, splashing with the act of coughing, and occasionally metallic tinkling. The two series of signs may occur in alternation. Both are incident to pulmonary cavities, tuberculous or otherwise, inclusive of pouch-like dilitation of the bronchial tubes; and, also, in the pleural space, in connection with perforation, or, in other words, in pneumo-hydrothorax.

"In the latter affection, the cavernous respiration oftener pre-

---

"*Under the head of Correlation of Physical Signs, I design to embrace only those which sustain toward each other direct relations. The signs incident to pleuritic effusion in the instances in which bronchial respiration exists over the compressed lung, are indirectly related, and, therefore, not included among those to which the term correlative is applied. For the same reason, I do not enumerate among correlative signs those supplementarily induced by various affections in parts of the lungs more or less remote from the situation of the disease.

sents the amphoric character; and the associated signs differ from those pertaining to pulmonary cavities. The percussion-resonance is more constantly tympanitic, is not circumscribed, but more or less diffused. Liquid, in greater or less quantity, is always present, and hence flatness co-exists with tympanitic resonance, the former situated above, and the latter below the level of the liquid. Metallic tinkling is generally observed, while in pulmonary cavities it is of rare occurrence. The succussion-sound is common, which is exceedingly infrequent in cavities formed within the lungs. The phenomena attendant on enlargement of the chest, are generally present in cases of pneumo-hydrothorax, and absent in intra-pulmonary excavations.

"7. *Adventitious Respiratory Sounds, or Rales.* — The adventitious sounds, or rales, may be considered under one heading, for, excepting a single species, viz., gurgling, they resemble each other in not sustaining toward other signs any fixed correlative relations. In this respect, they offer a striking contrast to the signs already enumerated. The moist and dry bronchial rales, including the sub-crepitant, generally represent pulmonary catarrh or bronchitis. They constitute all the positive or direct physical signs belonging to these affections. Other signs, it is true, are frequently found associated with them, but in such instances, pulmonary catarrh or bronchitis are superadded to other affections. The connection is one of coincidence, not of a pathological relation. This deficiency of correlative signs has a positive and important bearing on diagnosis. The presence of the bronchial rales, taken in connection with the absence of abnormal percussion-sound, or other signs, establishes the existence of the diseases which they represent, disconnected from other affections. The crepitant rale represents, in the great majority of the instances in which it is observed, pneumonitis. Pneumonitis during its career presents, as has been seen, a group of correlative signs; but the crepitant rale, strictly speaking, cannot be considered to stand in a correlative relation to any of them, for it is developed often prior to their appearance, and although it very frequently persists after other signs have appeared, this is by no means uniformly the case. Moreover, in a certain proportion of cases, it does not appear during the course of the affection. In the instance of this disease, as of bronchitis, the absence of co-existing signs is an important point, for, in connection with certain symptoms, it may denote the existence of pneumonitis, not advanced sufficiently to give rise to the pathological changes represented by associated signs. This point may have a materia influence on the therapeutical management of the disease. The indeterminate rales, although often combined with other physical phenomena, and deriving much of their diagnostic significance from the combination, have, nevertheless, no fixed or definite correlative signs. In other words, there are no signs involving the co-existence, even in a considerable proportion of instances only, of the indeterminate rales.

"8. *Friction Sounds.*—These resemble the foregoing rales in not sustaining definite correlative relations to other signs. They differ, however, in this respect, viz., clinically, they are very rarely found isolated; they are associated with signs to which they do not stand in a fixed or uniform relation. The associated signs are different, according to the different circumstances under which the friction-sounds are developed. Representing, in the great majority of instances, pleuritis, they may or may not be associated with the physical evidences of a certain amount of liquid effusion. Occurring either at the commencement or at a late period in the career of the disease, they may or may not be accompanied by the phenomena pertaining to contraction of the chest. Being incident not only to simple pleurisy, but occasionally to pleurisy developed as a complication of tuberculosis and pneumonitis, they may be found in combination with the groups of the physical signs representing the latter affections."

This chapter closes Part I. Part II., treating of "Diagnosis of Diseases affecting the Respiratory Organs," is equal or superior in interest to the previous part of the work, but we have no space for further comments or extracts. The analytical method has been pursued so far as practicable. We unhesitatingly commend the book to all who wish to become well acquainted with thoracic diseases, and the signs by which they may be distinguished. J.

